# Towards understanding paleoclimate impacts on primate de novo genes

**DOI:** 10.1093/g3journal/jkad135

**Published:** 2023-06-14

**Authors:** Xiao Liang, Lenwood S Heath

**Affiliations:** Department of Computer Science, Virginia Polytechnic Institute and State University, Blacksburg, VA 24061, USA; Department of Computer Science, Virginia Polytechnic Institute and State University, Blacksburg, VA 24061, USA

**Keywords:** de novo genes, primate genes, gene emergence, gene evolution, paleoclimate

## Abstract

De novo genes are genes that emerge as new genes in some species, such as primate de novo genes that emerge in certain primate species. Over the past decade, a great deal of research has been conducted regarding their emergence, origins, functions, and various attributes in different species, some of which have involved estimating the ages of de novo genes. However, limited by the number of species available for whole-genome sequencing, relatively few studies have focused specifically on the emergence time of primate de novo genes. Among those, even fewer investigate the association between primate gene emergence with environmental factors, such as paleoclimate (ancient climate) conditions. This study investigates the relationship between paleoclimate and human gene emergence at primate species divergence. Based on 32 available primate genome sequences, this study has revealed possible associations between temperature changes and the emergence of de novo primate genes. Overall, findings in this study are that de novo genes tended to emerge in the recent 13 MY when the temperature continues cooling, which is consistent with past findings. Furthermore, in the context of an overall trend of cooling temperature, new primate genes were more likely to emerge during local warming periods, where the warm temperature more closely resembled the environmental condition that preceded the cooling trend. Results also indicate that both primate de novo genes and human cancer-associated genes have later origins in comparison to random human genes. Future studies can be in-depth on understanding human de novo gene emergence from an environmental perspective as well as understanding species divergence from a gene emergence perspective.

## Introduction

In the past decade, many studies have been conducted on investigating de novo genes in yeast (Cai *et al.* 2008), nematodes (Prabh and Rödelsperger 2019), Drosophila (Reinhardt *et al.* 2013; Schlötterer 2015; Lu *et al.* 2018), mouse (Schmitz *et al.* 2018), and human (Blomen *et al.* 2015). Estimation of de novo gene emergence time has been a topic of interest and was investigated in many previous work (Beckenbach *et al.* 1993; Tautz and Domazet-Lošo 2011; Palmieri *et al.* 2014; Wilson *et al.* 2017; Baalsrud *et al.* 2018). However, due to the limited availability of whole-genome sequences, studies specifically addressing gene emergence in primate species were comparatively uncommon (Wu *et al.* 2011; Blomen *et al.* 2015; Ruiz-Orera *et al.* 2015). Among those, none investigate the association between primate gene emergence with paleoclimate (ancient climate) conditions. Similarly, studies on paleoclimate conditions rarely investigate primate species from a gene perspective.

As sequencing technology has advanced recently, there are an increasing number of whole-genome sequences available for various primate species (Sayers *et al.* 2021; Cunningham *et al.* 2022). This study leverages this information, combined with knowledge from literature on paleoclimate conditions, to explore various environmental conditions when primate de novo genes first emerged in primate species. Our investigation pointed specifically towards a preliminary comprehension regarding the association of paleoclimate with species divergence and gene evolution. The hope is that this study will provide an initial step to bridge the studies of paleoclimatology and of de novo gene emergence, with more future studies proceeding from both perspectives.

It is challenging, however, to obtain well-preserved genomes of ancestor primate species and to understand their genes directly (Hofreiter *et al.* 2015). Apart from the challenge of recovering DNA information from fossils of primate ancient species due to their old age, another contributing factor is the warm and humid environment many ancient primate species lived in, which is not ideal for DNA preservation. To understand how genes evolved in primates, an alternative is to construct evolutionary relations from present primate species, such as a timetree (Hedges *et al.* 2006), a tree that can be constructed from a group of species, depicting their evolutionary relationships and estimating their divergence time. In this study, 32 extant primate species (including human) with available whole-genome sequences were used to construct a timetree. The divergence time of different primate clades ranges from approximately 74 MYA, when primates first emerged, to approximately 2 MYA, when certain closely related species diverged from each other.

Genes studied in this work fall into three categories: (1) primate or human de novo genes identified in previous work (Evans *et al.* 2006; Li *et al.* 2010; Wu *et al.* 2011; Xie *et al.* 2012; Suenaga *et al.* 2014; Wang *et al.* 2014; Chen *et al.* 2015; Ruiz-Orera *et al.* 2015; Zhang *et al.* 2015; McLysaght and Hurst 2016; Kalebic *et al.* 2018; Suzuki *et al.* 2018; Sun *et al.* 2020; Yang *et al.* 2020; Saber *et al.* 2021), (2) human cancer-associated genes (Sondka *et al.* 2018) that are important to human health, and (3) random human genes as a comparison to the two nonrandom gene sets. Results show that primate or human de novo genes and human cancer-associated genes both have later origins compared to a random set of human genes. Moreover, these genes tend to emerge more frequently in recent years, especially from 13 MYA to the present, when the global temperature continues cooling. This finding is consistent with previous work (Tautz and Domazet-Lošo 2011). Furthermore, primate genes are likely to emerge during LWPs (local warming periods) in the context of an overall cooling trend, where an LWP is defined as a global surface temperature change greater than 0.5°C lasting for 0.25 MY. The LWPs more closely resemble the temperature condition that preceded the cooling trend and could be preferred by primates.

### De novo genes

De novo genes, as the name suggests, commonly refer to genes that are unique to a species or lineage. De novo genes can be detected by comparing genome sequences within or between species. During the past decade, more and more de novo genes have been identified and reported in different species, including yeast (Cai *et al.* 2008), nematodes (Prabh and Rödelsperger 2019), Drosophila (Reinhardt *et al.* 2013; Schlötterer 2015; Lu *et al.* 2018), mouse (Schmitz *et al.* 2018), primates (Wang *et al.* 2014), hominoids (Knowles and McLysaght 2009; Xie *et al.* 2012; Suenaga *et al.* 2014), and human (Blomen *et al.* 2015). Questions behind these genes, such as their origins and ages, have been a topic of interest in research over the past few years.

So far, there have been two major mechanisms proposed for de novo gene emergence: (1) gene duplication (Tautz and Domazet-Lošo 2011; Florio *et al.* 2015; Dougherty *et al.* 2017) and (2) emergence from noncoding regions (Heinen *et al.* 2009; Knowles and McLysaght 2009; Carvunis *et al.* 2012; Xie *et al.* 2012; Reinhardt *et al.* 2013; Suenaga *et al.* 2014; Wang *et al.* 2014; Zhao *et al.* 2014; Prabh and Rödelsperger 2019). Both mechanisms have abundant evidence in various species. The latter is more associated with species divergence. A coding sequence in one species can be a homolog to a noncoding region in another related species. One speculated mechanism is that both species inherited the noncoding region from a common ancestor, while only in some species this region evolved into a gene (Knowles and McLysaght 2009).

In various species, some de novo genes are reported to have important roles. For example, human-specific genes ARHGAP11B and NOTCH2NL are reported to be associated with neocortical expansion in the human brain (Florio *et al.* 2017; Kalebic *et al.* 2018; Suzuki *et al.* 2018; Sun *et al.* 2020). Essential de novo genes are also reported in other species, including yeast (Cai *et al.* 2008), Drosophila (Reinhardt *et al.* 2013), primates (Wang *et al.* 2014), and human (Blomen *et al.* 2015). On the other hand, some de novo genes are shown to be associated with diseases, including cancer (Vakirlis *et al.* 2020), Alzheimer’s disease (Chen *et al.* 2015), primary microcephaly (Evans *et al.* 2006), and neuroblastoma (Suenaga *et al.* 2014).

In addition to de novo genes being associated with human diseases, research has also shown that primate-specific genes (i.e. primate de novo genes) can contribute to human diseases, such as cancer. For example, Ma *et al.* (2022) reported that certain primate-specific genes are upregulated in 13 cancer types, possibly facilitated by copy-number gain and promoter hypomethylation. It is also notable that human cancer research has been long conducted on model species such as mouse. However, not all human cancers can be effectively studied in mouse. The two species do not share all cancer types, and certain human cancer-associated genes do not have detected homologs in mouse, rat, or dog (Khanna *et al.* 2006; Bürtin *et al.* 2020). Therefore, tumor-bearing monkeys (TBMs) and nonhuman primates (NPHs) were investigated for cancer studies (Dumont *et al.* 2004; Xia and Chen 2011). Here, a possible link between primate de novo genes and cancer-associated genes has been indicated but has not been further investigated in previous work. Given the unsuitability of rodent or dog models on studying certain cancer genes, investigation on primate de novo genes and cancer-associated genes can potentially provide special insights on understanding human cancer.

Few studies have been conducted on associating the environmental conditions regarding *de novo* gene emergence. One study on codfish shows an antifreeze associated de novo gene *afgp* is lost in a specific non-Antarctic lineage, implying a possible link between environmental conditions and de novo gene loss (Baalsrud *et al.* 2018). Tautz and Domazet-Lošo (2011) investigated specifically paleoclimate conditions. They have shown that the emergence of mouse genes peaked in two time ranges. One of them is during the recent 50 MYA, which is the finest resolution for recent years in this work. The other is approximately 800 MYA, when Earth was undergoing several freezing cycles. For the 800 MYA case, these genes originally emerged in ancient species and survive today in mouse.

### Evolutionary history of primate species

To understand the environmental conditions under which primate genes emerged, it is necessary to first know the evolutionary history of the primate species. The earliest primate fossil record found so far is between 65 and 55 MYA (Bloch *et al.* 2007), where the latter is around the boundary between the Paleocene and the Eocene. Due to the scarcity of fossils for certain areas and extinct species, some studies infer the divergence time of the common ancestor of primates, using molecular clock and phylogeny methods (Fabre *et al.* 2009; Springer *et al.* 2012; Tiley *et al.* 2020; Upham *et al.* 2021). Through these methods, the first appearance of the primate common ancestor is speculated to be approximately 74 MYA, with an estimated range from 71 to 77 MYA. One explanation on this difference is that fossil evidence generally underestimates the species divergence time (Koch 1978; Reisz and Müller 2004; Steiper and Young 2008).

Although the two approaches give different divergence times, previous studies associated with paleoclimate conditions have been based mainly on the one supported by fossil evidence, which is approximately 65–55 MYA. Approximately 66 MYA, a mass extinction event K-T (Cretaceous-Paleocene extinction event) (Arreguín-Rodríguez *et al.* 2018; Keller *et al.* 2018) caused the loss of most of the existing species on Earth (Jablonski 1994). Another mass extinction event of benthic foraminifera occurring approximately 56 MYA has been linked to PETM (Paleocene–Eocene thermal maximum), an extremely rapid environmental warming event (Bowen *et al.* 2015). During PETM, the global temperature increased 5–8°C in about 200,000 years (Gingerich 2006; McInerney and Wing 2011). The warmer temperature on Earth caused by PETM, together with another warm period EECO (Early Eocene Climatic Optimum) from approximately 52 to 50 MYA (Zachos *et al.* 2001), is suggested to have provided an ideal environment for the appearance and prosperity of early primates (Gingerich 2012).

Warm climate was not the only weather pattern affecting the evolution of primates. Approximately 48 to 33.5 MYA, the latter half of Eocene has endured a long cooling trend (Bijl *et al.* 2009; Hollis *et al.* 2009, 2012; Westerhold *et al.* 2020). This gradual cooling trend was followed by a precipitous fall both in temperature and sea level, namely the TEE (Terminal Eocene Event) approximately 34 MYA (Van Couvering *et al.* 1981). TEE is suggested to be associated with another mass extinction event, namely the Eocene–Oligocene extinction event (Prothero 1985, 1994). This rapid change in temperature not only forced the primates of the time to move to new habitats (Barry *et al.* 2002; Eronen and Rook 2004), but also changed the ecology of the habitats themselves, including the composition of the forests (Collinson and Hooker 1987; Janis 1993; Jablonski 2005).

Hominoids and Old World monkeys first appeared approximately 25–23 MYA around the beginning of the Miocene (Goodman *et al.* 1998; Stauffer *et al.* 2001). At that time, the environmental conditions became rather drier and the temperature increased again, although not to Early Eocene levels. A steeper latitudinal thermal gradient was also formed (Savin *et al.* 1985; Pound *et al.* 2012). Miocene Climatic Optimum, another warm period from about 18 to 14 MYA (Bohaty *et al.* 2009), preceded the Middle Miocene extinction event (Raup and Sepkoski 1986; Böhme 2003). After that, starting from 13 to 12 MYA, the temperature again began a consistent cooling trend continuing to the present day (Herbert *et al.* 2016). This cooling trend is associated with the Late Miocene faunal turnover, which refers to the simultaneous appearance and disappearance of species from a community (Badgley and Gingerich 1988). The even drier and seasonal environments only started becoming closer to present climate approximately 9.2 MYA (Barry *et al.* 2002). Loss of evergreen forests caused both the extinction of several taxa of primates in Europe, referred to as the Vallesian Crisis (Casanovas-Vilar *et al.* 2005), and the expansion of Old World monkeys during 6–7 MYA (Jablonski *et al.* 2000).

However, it is difficult to obtain ancient nonhuman primate genomes and investigate their genes directly. First of all, the preservation and retrieval of ancient DNA in general tend to be challenging, with the oldest known sample dating back approximately a million years (Willerslev *et al.* 2004). For primates, the situation is no less difficult as the environmental conditions of primate habitats were not ideal for DNA preservation. Hofreiter *et al.* (2015) showed that ancient nonhuman primates tended to live in environments of high temperature, high humidity, and acidic soil. Estimating the gene sequences of ancient primate species based on their present descendants is one approach to mitigate the problem. However, as previously mentioned, these analytical studies of gene emergence were rarely combined with paleoclimatic studies on the environmental conditions of speciation. It is even more so in the topic of de novo gene emergence. This study is intended to offer a small step to bridge between the two areas to inspire future work.

## Materials and methods

There are four types of data used in this work: (1) a timetree for 32 primate species (Kumar *et al.* 2022), (2) protein sequence data of 145 select and 240 random primate genes (Evans *et al.* 2006; Li *et al.* 2010; Wu *et al.* 2011; Xie *et al.* 2012; Suenaga *et al.* 2014; Wang *et al.* 2014; Chen *et al.* 2015; Ruiz-Orera *et al.* 2015; Zhang *et al.* 2015; McLysaght and Hurst 2016; Kalebic *et al.* 2018; Sondka *et al.* 2018; Suzuki *et al.* 2018; Sun *et al.* 2020; Yang *et al.* 2020; Saber *et al.* 2021; The UniProt Consortium 2021), (3) CDS (coding sequence) data of 32 primate species (Sayers *et al.* 2021; Cunningham *et al.* 2022), and (4) temperature data for the past 66 MY (Hansen *et al.* 2013). Supporting data include geological distribution of the primate species and occurrence time of major geological events.

To detect genes in primate species, protein sequences of these genes were aligned with the coding sequences of primate species. After applying identity and coverage thresholds to the BLAST results, each gene was assigned to be present or absent in each primate clade on the timetree constructed from the 32 primate species (Kumar *et al.* 2022). Based on the status of presence or absence in each primate clade, an emergence time was estimated for each gene based on the clade divergence time. The temperature curve for the past 66 MY (Hansen *et al.* 2013) was then aligned with the divergence time points to draw further conclusions.

### Timetree of primate species

From Ensembl and NCBI databases (Sayers *et al.* 2021; Cunningham *et al.* 2022), whole-genome and CDS (coding sequence) sequence data were downloaded for 32 nonduplicated and available primate species (including human, *Homo sapiens*), as shown in Table 1. Out of the 32 primate species, the divergence times and ranges of 31 species (excluding *Cebus imitator*) were obtained from the TimeTree database (Kumar *et al.* 2022). These divergence times and ranges on TimeTree were synthesized and derived from studies (Hedges *et al.* 2015; Kumar *et al.* 2022).

**Table 1.** Distribution of 31 nonhuman primate species used in this research.

Species	Distributed area (Island)
*Otolemur garnettii*	East Africa
*Prolemur simus*	Africa (Madagascar)
*Lemur catta*	Africa (Madagascar)
*Propithecus coquereli*	Africa (Madagascar)
*Microcebus murinus*	Africa (Madagascar)
*Callithrix jacchus*	South America
*Saimiri boliviensis*	South America
*Aotus nancymaae*	South America
*Cebus imitator*	Central America
*Cebus capucinus*	Central America and South America
*Sapajus apella*	South America
*Hylobates moloch*	Southeast Asia (Java)
*Nomascus leucogenys*	Southeast Asia
*Gorilla gorilla*	Central Africa
*Pan paniscus*	Central Africa
*Pan troglodytes*	Sub-Saharan Africa
*Pongo abelii*	Southeast Asia (Sumatra)
*Chlorocebus sabaeus*	West Africa
*Mandrillus leucophaeus*	West Africa
*Cercocebus atys*	West Africa
*Papio anubis*	West Africa, Sahara, and Sub-Saharan Africa
*Theropithecus gelada*	Horn of Africa
*Macaca mulatta*	South, Central, and Southeast Asia
*Macaca fascicularis*	Southeast Asia and Oceania
*Macaca nemestrina*	Southeast Asia
*Piliocolobus tephrosceles*	Africa
*Colobus angolensis*	Central Africa
*Trachypithecus francoisi*	Southeast Asia
*Rhinopithecus roxellana*	East Asia
*Rhinopithecus bieti*	East Asia
*Carlito syrichta*	Southeast Asia

**Fig. 1.**
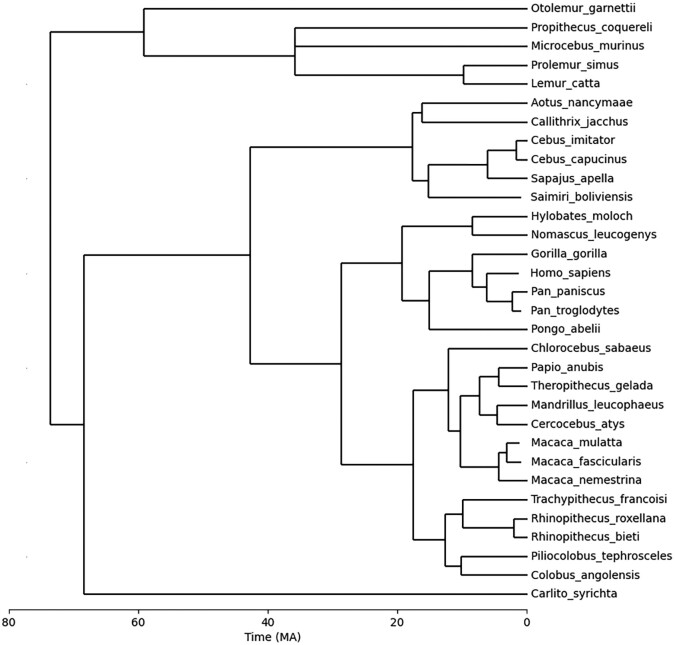
A timetree of 32 primate species. The node and branch of *Cebus imitator* was obtained from Boubli *et al.* (2012) and integrated into the timetree. Divergence times of the other 31 species were obtained from the TimeTree database (Kumar *et al.* 2022). The confidence interval for divergence times on TimeTree is 95%, indicating that approximately 95% of reported times in the synthesized studies lie within 2 SDs of the mean time, assuming a normal distribution.

Species are sorted according to their order in the timetree as shown in Figure 1. Most of these 31 primate species are distributed in Africa, South America, and Southeast Asia.

The primate species *Cebus imitator* was not found in the TimeTree database (Kumar *et al.* 2022). Its divergence time from another species *Cebus capucinus*, 1.7 MYA, was separately obtained from Boubli *et al.* (2012). Figure 1 shows the integrated timetree of the 32 primate species drawn using the Biopython library (Cock *et al*. 2009) in Python 3.10.8.

TimeTree is a resource that has been widely used in previous study regarding de novo genes (Moyers and Zhang 2015; Schmitz *et al.* 2018; Dowling *et al.* 2020; Heames *et al.* 2020). As a synthesized tree, we consider it more informative and less biased than a single tree from a sole source, providing a confidence interval for the readers’ reference. The TimeTree’s confidence interval is set at 95%, indicating that approximately 95% of reported times in the synthesized studies lie within 2 SDs of the mean time, assuming a normal distribution. On the timetree obtained from the TimeTree database, certain clades share a common ancestor node with a widely recognized taxonomic name. For instance, in the present study, Colobinae serves as the subfamily name for the following species: {*Piliocolobus tephrosceles, Colobus angolensis palliatus, Trachypithecus francoisi, Rhinopithecus roxellana*, and *Rhinopithecus bieti*}. Note that this is not a comprehensive set under the subfamily when considered outside the context of this research. The widely recognized taxonomic name indicates the divergence time of that node, such as the subfamily node *Colobinae*, was likely directly derived from literature instead of inferred from other nodes on the tree. In this study, these nodes were considered comparatively more reliable than those without a taxonomic name.

### Detecting genes in primate CDS

There are three sets of human genes investigated in this article: (1) 45 primate or human de novo genes (Evans *et al.* 2006; Li *et al.* 2010; Wu *et al.* 2011; Xie *et al.* 2012; Suenaga *et al.* 2014; Wang *et al.* 2014; Chen *et al.* 2015; Ruiz-Orera *et al.* 2015; Zhang *et al.* 2015; McLysaght and Hurst 2016; Kalebic *et al.* 2018; Suzuki *et al.* 2018; Sun *et al.* 2020; Yang *et al.* 2020; Saber *et al.* 2021), (2) 100 randomly selected human cancer-associated genes out of 733 available (Sondka *et al.* 2018), and (3) 240 randomly selected human genes using random.sample() function in Python 3.10.8, with a splitting into 60 and 180 in result analysis as detailed in “Results and discussion.” Protein sequences of these 385 genes were obtained from UniProt (The UniProt Consortium 2021). Primate or human de novo genes are the major motivating subject to study in this article and are supposed to have emerged recently within the evolutionary time range of primates. In the previous work (Khanna *et al.* 2006; Bürtin *et al.* 2020; Ma *et al.* 2022), cancer-associated genes have been indicated to have some association with primate-specific genes (i.e. primate de novo genes), such as certain primate-specific genes are upregulated in some cancer types (Ma *et al.* 2022). Therefore in this study, cancer-associated genes are investigated along with the primate or human de novo genes to further understand their relation, as well as to provide some insights on cancer genes from the de novo gene perspective. Here, a subset of human genes was processed rather than the complete set due to computational resource constraints. As a reference, aligning 240 genes across 32 species takes approximately 1 week on our server machine.

Primate or human de novo genes were identified in a number of papers (Evans *et al.* 2006; Li *et al.* 2010; Wu *et al.* 2011; Xie *et al.* 2012; Suenaga *et al.* 2014; Wang *et al.* 2014; Chen *et al.* 2015; Ruiz-Orera *et al.* 2015; Zhang *et al.* 2015; McLysaght and Hurst 2016; Kalebic *et al.* 2018; Suzuki *et al.* 2018; Sun *et al.* 2020; Yang *et al.* 2020; Saber *et al.* 2021). Specifically, the original storage repository for data from Wu *et al.* (2011) was not accessible in June 2022, and the data table was acquired from Xie *et al.* (2012), which cited and organized the data from the previous paper. Based on these genes, 46 available protein sequences are identified in UniProt (The UniProt Consortium 2021) records. Notably, the gene *SRGAP2* is mapped to two reviewed proteins, both of which were detected across all 32 primate species, therefore do not affect the results as they are estimated to have emerged beyond the recent 66 MY. For random selected human genes, there is one gene *FAM47E-STBD1* not mapping to any UniProt protein ID. Additionally, several genes are mapped to multiple reviewed proteins. In these cases, we only considered the first protein sequence listed in the UniProt (The UniProt Consortium 2021) ID mapping table. The full list of human genes to randomly select from is extracted from the human CDS sequence (Genome Reference Consortium Human Build 38) on Ensembl (Cunningham *et al.* 2022), data and scripts available in the repository described in “Data availability.”

Human cancer-associated genes were obtained from the Cancer Gene Census (Sondka *et al.* 2018), version 96. Among 733 available cancer-associated genes, 100 genes that do not overlap with the primate or human de novo gene set were randomly selected for this project, for both computational resource conservation and data set balance (i.e. not to allow the results be overly biased by cancer genes).

BLAST (Altschul *et al.* 1990), namely the TBLASTN method that searches proteins against nucleotides, was used to detect similar coding sequences in the 31 nonhuman primate species. Nucleotide references were built locally from the CDS data of 32 primate species. The 385 genes were then searched against the references with default parameters. Results were post-filtered with thresholds of 80% identity and 80% subject coverage.

The advantage of BLAST is it can handle large scale experiments. BLAST is shown to provide quality results and has been used for the purpose of finding de novo genes in recent work (Heames *et al.* 2020; Rivard *et al.* 2021). There are also some limitations that have been recognized, such as the basic BLAST methods do not support sliced alignment, which maps a single sequence to multiple split reference regions (Boratyn *et al.* 2019). Some previous work considered it necessary to provide evidence other than BLAST to identify de novo genes (Tautz and Domazet-Lošo 2011). In the case of this study, since it aims at understanding the association of paleoclimate conditions with gene emergence, not declaring certain de novo gene emergence time or finding new de novo genes, the BLAST approach was considered adequate.

### Assigning genes to primate clades

On the constructed timetree, the 32 present species were split into clades at different taxonomic levels. Here, we follow the standard definition of a clade, where a clade is a group of species that share a common ancestor. This group can vary in size. For each clade, each gene was assigned to one of three statuses: (1) existent, (2) absent, and (3) unknown. A gene was only assigned to exist in one clade, if this gene was detected in more than 80% species in that clade. A gene was marked to be absent from one species clade, if this gene was not detected in more than 80% species in that clade. If neither of the above, there was not sufficient information to assign a status other than unknown. The thresholds of 80% identity and 80% subject coverage on BLAST results were applied before assigning the genes to clades.

There are two reasons for setting the 80% thresholds in this approach: (1) mitigating the bias introduced by the inevitable inaccuracy in the detection method and (2) the preference to accept a common algorithm used in phylogenetic tree construction (Capra *et al.* 2013), namely the Wagner parsimony (Kluge and Farris 1969). In terms of gene emergence, Wagner parsimony assumes that the status of a gene is reversible. This algorithm was chosen over another prevalent one, namely Dollo parsimony (Gould 1970), which assumes a gene can only be gained one time and cannot be regained if lost. Wagner parsimony was preferred in this study for there were genes reported in codfish (Baalsrud *et al.* 2018) and plants (Esfeld *et al.* 2018) to have revived from noncoding sequences, which supports Wagner parsimony rather than Dollo parsimony.

After the genes were assigned to different primate clades, timetree nodes were identified when a gene was present in one child branch but was absent in the other. Based on divergence times given by the TimeTree database (Kumar *et al.* 2022), the emergence time of that gene was then estimated to be the divergence time of such a node (i.e. the divergence time of the two children clades). When multiple possible divergence times were suggested, the earliest one was utilized.

### Temperature curve

Temperature data up to 66 MYA was obtained from Hansen *et al.* (2013). In this work, global surface temperature $T_{s}$ was estimated from deep ocean temperature $T_{do}$. The deep ocean temperature $T_{do}$ was yielded from the oxygen isotope ratio (δ18O related to δ16O) in foraminifera shells, a commonly used proxy for ancient temperature. The original data has an uneven density of data points at different time points (i.e. density increases as time approaches the present), which was kept unchanged in this article. The temperature curve and related analysis in this article were based on the global surface temperature $T_{s}$.

## Results and discussion

The results and corresponding discussion of this study unfold in four directions: (1) the emergence time of de novo genes compared to human cancer-associated genes, (2) the temperature conditions at primate gene emergence, (3) other environmental conditions at primate gene emergence, and (4) limitations and possible future improvements on this study.

### Similarity between de novo genes and cancer-associated genes

As introduced in “Introduction,” previous literature has suggested a potential association between primate de novo genes and human cancer-associated genes. In this study, we demonstrate that from a gene emergence frequency perspective, primate *de novo* genes may exhibit similarities to human cancer-associated genes.

**Fig. 2.**
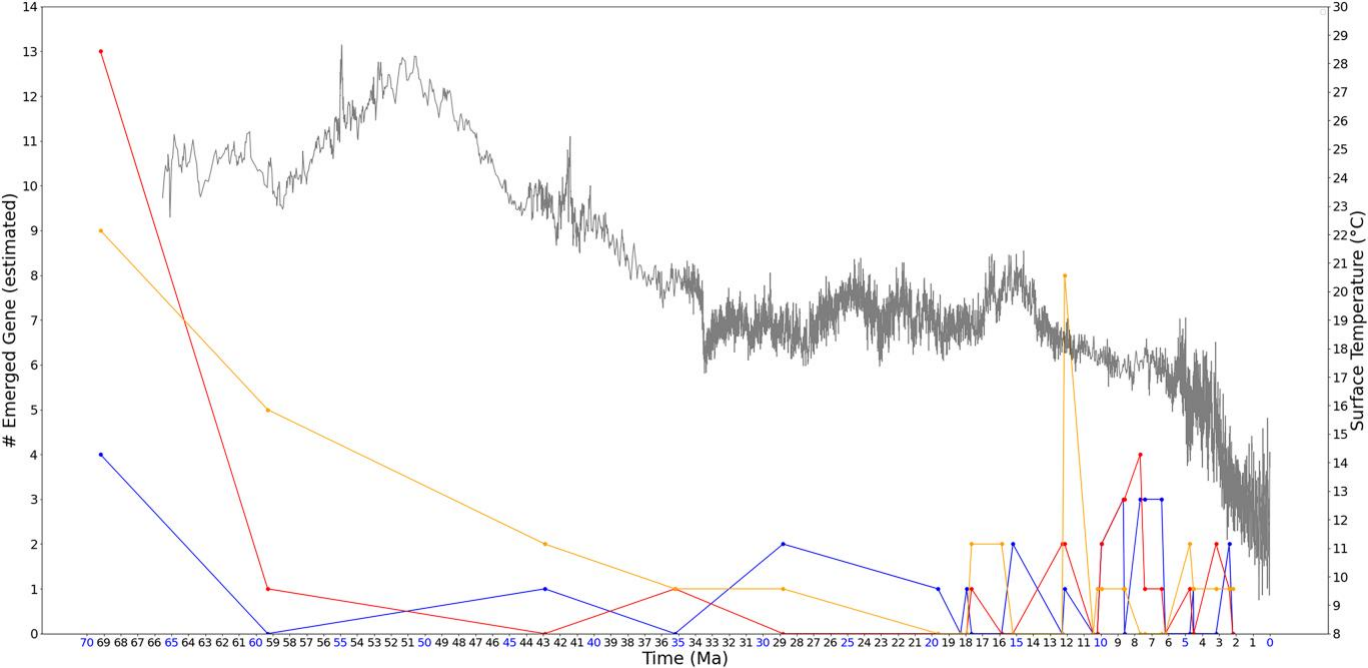
Estimated number of emerged human genes overlayed on temperature change associated with time. Blue markers represent primate or human de novo genes obtained from Evans *et al.* (2006); Li *et al.* (2010); Wu *et al.* (2011); Xie *et al.* (2012); Suenaga *et al.* (2014); Wang *et al.* (2014); Chen *et al.* (2015); Ruiz-Orera *et al.* (2015); Zhang *et al.* (2015); McLysaght and Hurst (2016); Kalebic *et al.* (2018); Suzuki *et al.* (2018); Sun *et al.* (2020); Yang *et al.* (2020); Saber *et al.* (2021), red markers starting at 13 on the y-axis represent cancer-associated genes, orange markers starting at 9 on the y-axis represent a set of 60 random human genes as a comparison, and the gray curve represents the global surface temperature (°C) from 66 MYA to present. Compared to cancer-associated genes, primate or human de novo genes have more recent emergence times. Peaks of gene emergence occur in recent 13 MY, during the consistent cooling in Late Miocene that continues to today. Specifically, more than half of the nonrandom genes are estimated to have emerged within recent 10 MY. This result is consistent with Tautz and Domazet-Lošo (2011). The peak of emergence of random genes around 12 MYA occurs earlier than the nonrandom genes around 6–10 MYA, possibly indicating that literature reported de novo genes and cancer-associated genes both have later origins compared to general human genes.

Figure 2 depicts the time-dependent distribution of emerged genes. In this figure, de novo genes and cancer-associated genes are illustrated separately, with comparison to a random set of 60 human genes. In the illustrated distribution, several high peaks of gene emergence are observed within recent 13 MY, coinciding with the consistent cooling in the Late Miocene that continues to today. This trend is observed regardless of whether the gene set is random or nonrandom (i.e. primate de novo genes and cancer associated genes). However, compared to the peak at approximately 12 MYA for the random set of human genes, the peaks of primate de novo genes and human cancer-associated genes occur later, approximately 10–6 MYA. This finding possibly indicates an association or similarity between primate de novo genes and human cancer-associated genes. Relative to a random human gene set, both the primate de novo genes and human cancer-associated genes exhibit later origins (within the recent 13 MYA range) and share a similar peak of gene emergence time at approximately 10–6 MYA.

**Fig. 3.**
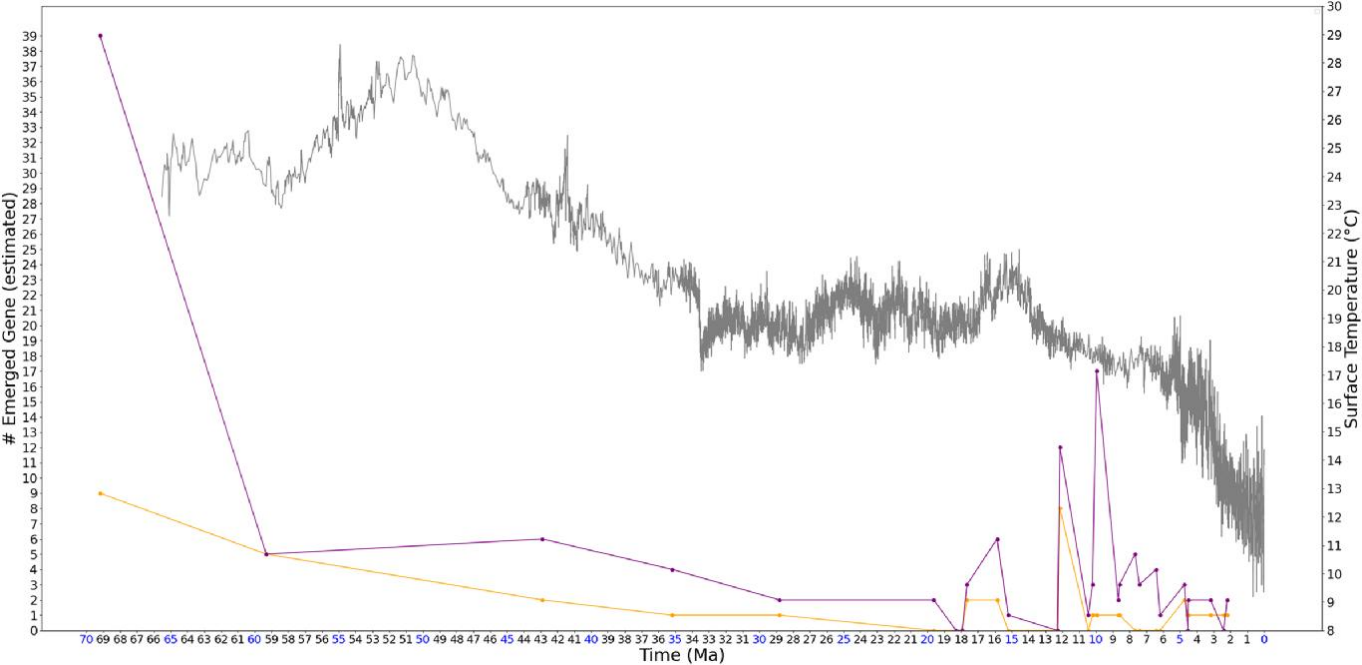
Estimated number of emerged human genes and temperature change associated with time. Purple markers represent the estimated number of emerged human genes in a 180 random gene set, orange markers represent the estimated number of emerged human genes in a 60 random gene set, and the gray curve represents the global surface temperature (°C) from 66 MYA to present. As illustrated, the emergence time of 180 random human genes has different peaks than the 60 random human genes, but the overall trend of emerging peaks (around 12 and 10–9 MYA) preceding the peaks of nonrandom genes is not affected by the size of random set.

This observation remains consistent when analyzing a larger random gene set of 180. In addition to comparing the 60 random gene set, we conducted another experiment using a set of 180 human genes randomly selected through random.sample() function in Python 3.10.8, following the same method used for the 60 random gene selection as described in “Materials and methods.” Due to time and resource constraints, we limited the analysis to a subset of human genes rather than the entire gene set, as aligning the 240 random genes across 32 species takes approximately 1 week on our server machine. The results for the 180 random human genes are not plotted together with the nonrandom gene sets, to avoid smoothing the peaks in the nonrandom gene sets due to the difference in data size. Instead, Fig. 3 displays the results for the 180 random human genes alongside the 60 random human gene set, using the latter as a reference for readers to visualize the relationship between the results of the 180 random genes and the nonrandom gene set. As illustrated in the figure, the emergence time of 180 random human genes has several different peaks than the 60 random gene set, but the overall trend of emerging peaks (at approximately 12 and 10–9 MYA) preceding the peaks of nonrandom genes remains unaffected by the random set size.

### Temperature conditions at primate gene emergence

#### Primate de novo genes emergence in local warm periods

From the 32 primate species (including human) and 145 nonrandom human genes processed, there were 67 genes found in the CDS of a proper subset of these primate species, with thresholds of 80% identity and 80% subject coverage identified using BLAST. Among the 67 emerged genes during the evolution of the 32 primate species, 38 of them are cancer-associated genes (out of 100 processed cancer-associated genes), and the other 29 are primate or human de novo genes reported in (Evans *et al.* 2006; Li *et al.* 2010; Wu *et al.* 2011; Xie *et al.* 2012; Suenaga *et al.* 2014; Wang *et al.* 2014; Chen *et al.* 2015; Ruiz-Orera *et al.* 2015; Zhang *et al.* 2015; McLysaght and Hurst 2016; Kalebic *et al.* 2018; Suzuki *et al.* 2018; Sun *et al.* 2020; Yang *et al.* 2020; Saber *et al.* 2021) (out of 45 processed de novo genes). The estimated emerging time of each gene was then assigned based on divergence times of primate clades, according to the gene occurrence in the two branches of the node on the phylogenetic tree. (For details, see “Detecting genes in primate CDS”.)

**Fig. 4.**
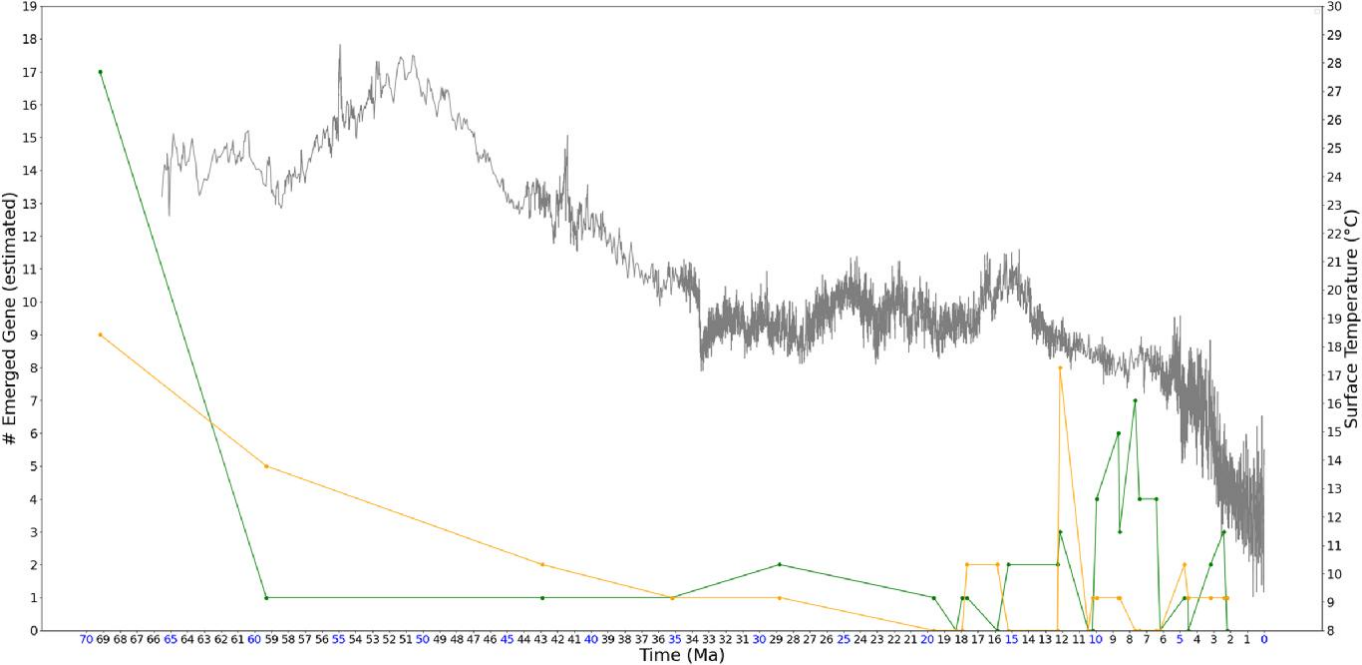
Estimated number of emerged human genes overlayed on temperature change associated with time. Note that the *y*-axis range is different from Fig. 2. Green markers represent primate or human de novo genes obtained from (Evans *et al.* 2006; Li *et al.* 2010; Wu *et al.* 2011; Xie *et al.* 2012; Suenaga *et al.* 2014; Wang *et al.* 2014; Chen *et al.* 2015; Ruiz-Orera *et al.* 2015; Zhang *et al.* 2015; McLysaght and Hurst 2016; Kalebic *et al.* 2018; Suzuki *et al.* 2018; Sun *et al.* 2020; Yang *et al.* 2020; Saber *et al.* 2021), as well as cancer-associated genes obtained from Cancer Gene Census (Sondka *et al.* 2018). Orange markers represent a random set of 60 human genes as a comparison to the nonrandom genes. The gray curve represents the global surface temperature (°C) from 66 MYA to present. Peaks of gene emergence occur in recent 13 MY, during the consistent cooling in Late Miocene that continues to today, being consist with Tautz and Domazet-Lošo (2011).

The time-dependent distribution of the total number of emerged genes is shown in Fig. 4, with a randomly set of 60 human genes serving as a comparative reference at an equivalent data size scale. In the illustrated distribution, several peaks of gene emergence occur within recent 13 MY, during the consistent cooling in the Late Miocene that continues to today. Specifically, more than half of the nonrandom genes (35 out of 67) are estimated to have emerged in the recent 10 MY. This is consistent with (Tautz and Domazet-Lošo 2011), where the emerging rates of orphan genes (de novo genes) in mouse, *Drosophila* and *Arabidopsis* are considered to be high in the recent 50 MY (with no finer time resolution). Also in this previous work, the emerging rate in mouse has peaked when the surface temperature was experiencing a freezing cycle period, namely the Snowball Earth (Hoffman *et al.* 1998).

**Fig. 5.**
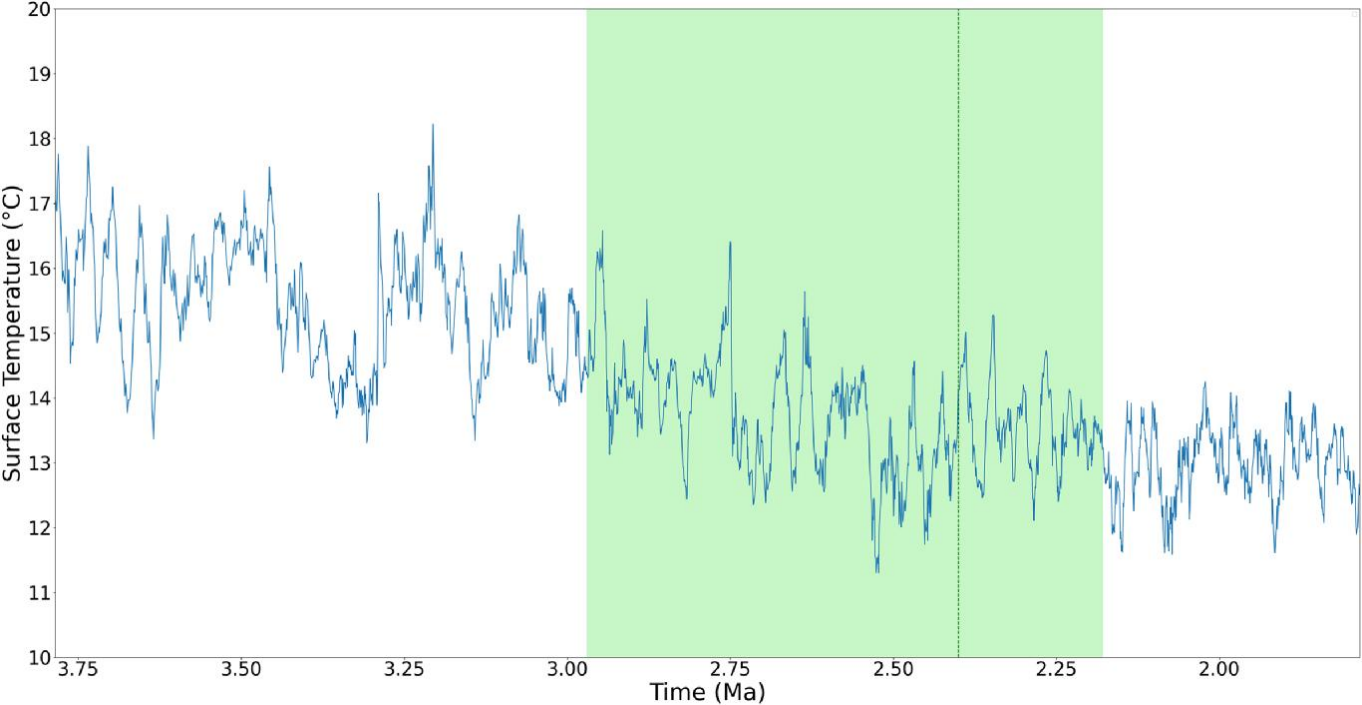
Estimated divergence time of genus *Pan*. The vertical dark green line shows the estimated divergence time, and the green area shows the estimated range of divergence time synthesized from literature. While the overall temperature is declining, the estimated species divergence and gene emerging time falls at local warming periods.

**Fig. 6.**
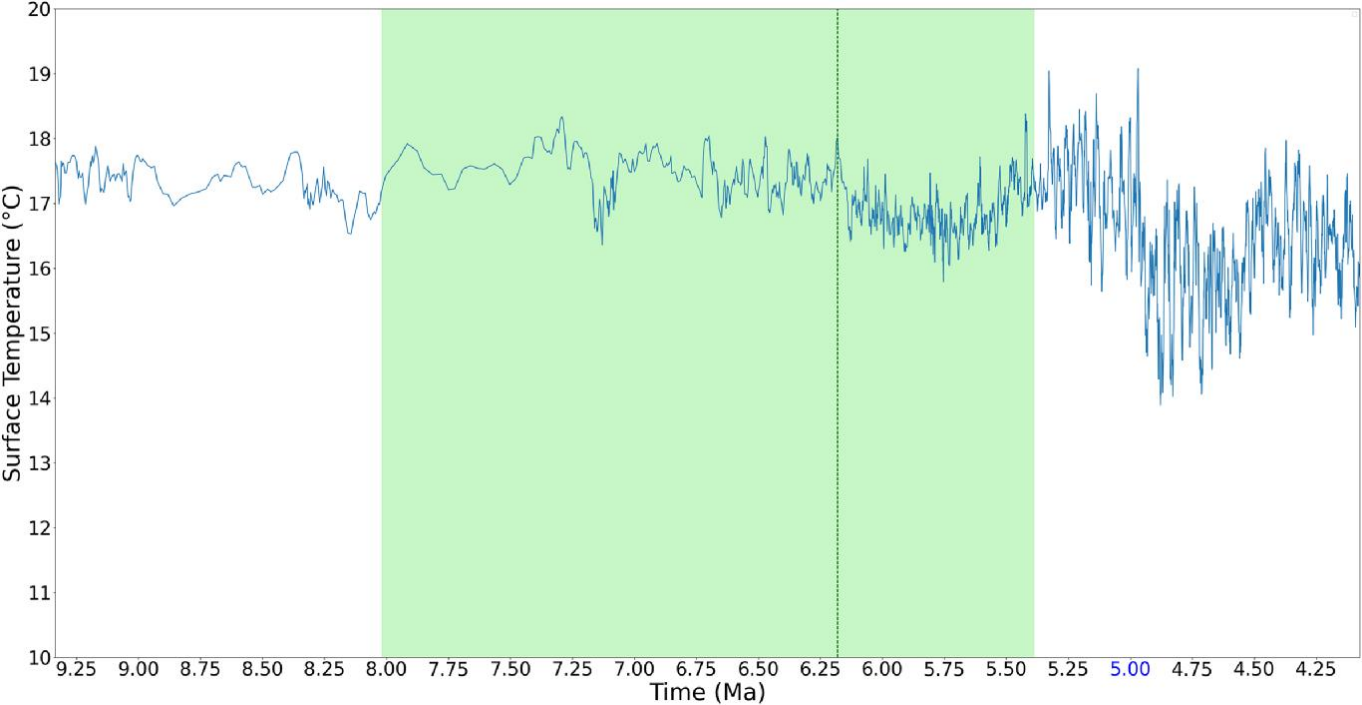
Estimated divergence time of subfamily *Cebinae*. The vertical dark green line shows the estimated divergence time, and the green area shows the estimated range of divergence time synthesized from literature. While the overall temperature is declining, the estimated species divergence and gene emerging time fall at local warming periods.

**Fig. 7.**
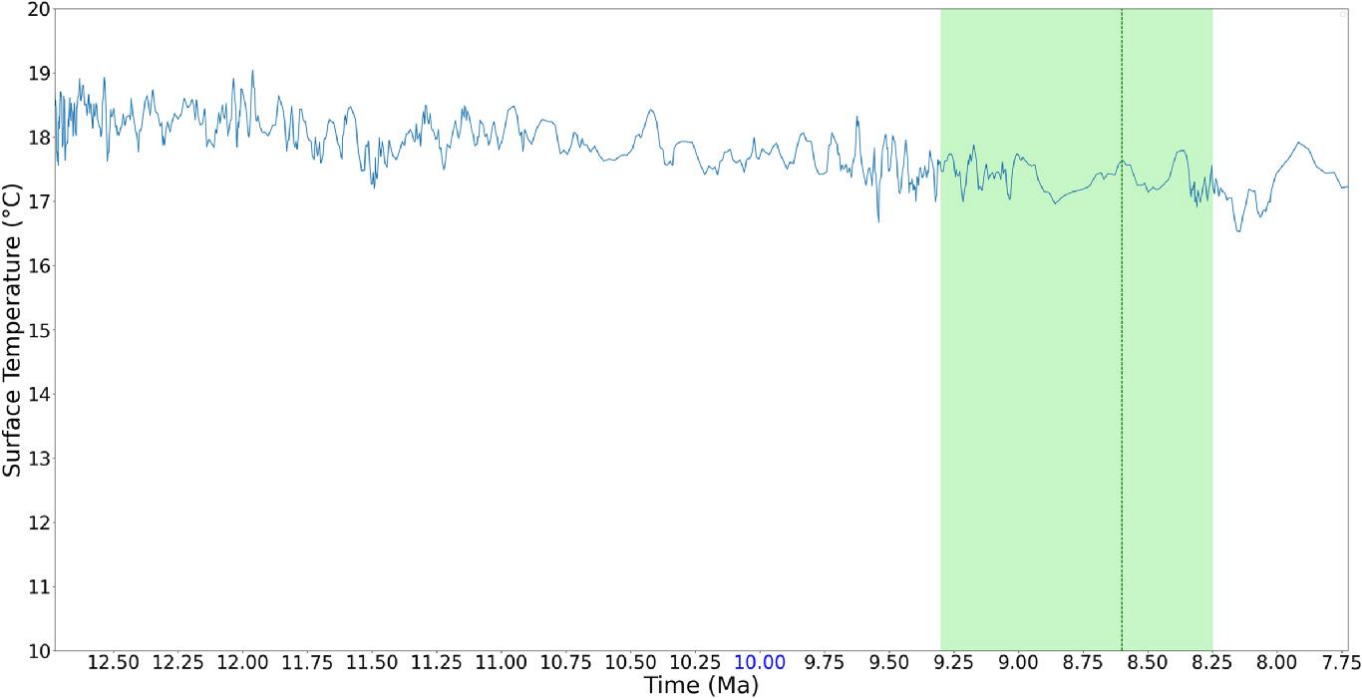
Estimated divergence time of subfamily *Homininae*. The vertical dark green line shows the estimated divergence time, and the green area shows the estimated range of divergence time synthesized from literature. While the overall temperature is declining, the estimated species divergence and gene emerging time fall at local warming periods.

**Fig. 8.**
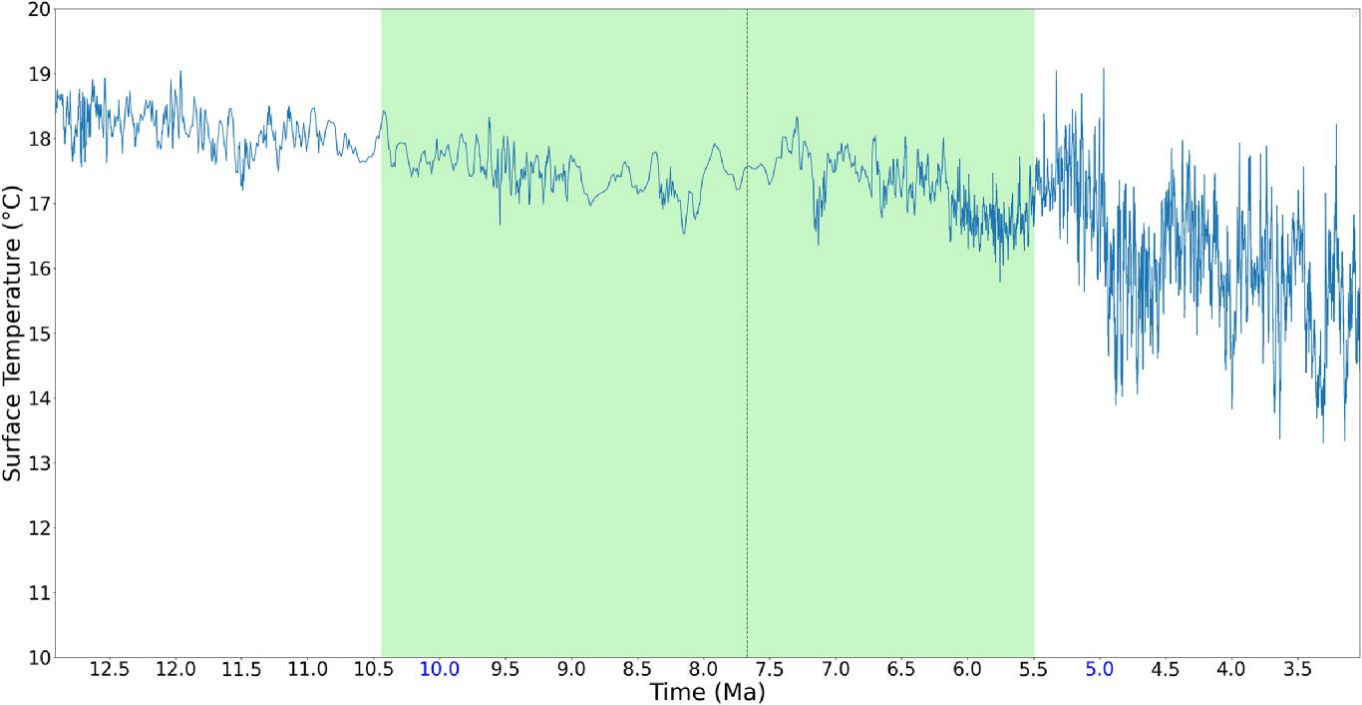
An estimated emerging time of 10 genes out of 77. This is the estimated divergence time between {*Trachypithecus francoisi*} and {*Rhinopithecus roxellana, Rhinopithecus bieti*}. The vertical dark green line shows the estimated divergence time, and the green area shows the estimated range of divergence time synthesized from literature. While the overall temperature is declining, the estimated species divergence and gene emerging time fall at local warming periods.

It is more interesting, however, if the individual regions of the peaks are zoomed in to observe. Figures 5–8 give four zoomed in regions within the recent 10 MY, including the three divergence times that have rank names (divergence time of genus *Pan*, subfamily *Cebinae*, and subfamily *Homininae*) and one divergence time approximately 7.67 MYA, which has a peak of 7 emerged genes. As observed in the zoomed in regions, although these genes are estimated to emerge during the cooling trend, these peaks fall at LWPs (local warming periods).

Previous work has suggested that in terms of ancient environment, temperature change on the order of 1°C that lasts on the order of million years can be considered a warming effect. Such a warm effect can cause environmental changes such as ice melting, sea level rising, and storms (Dunbar 1998; Brierley and Fedorov 2011; Hansen *et al.* 2016). Schneider (Schneider 1994) suggested that a century-long temperature change of 0.5°C on global surface is unlikely to be a totally natural climatic fluctuation. With such prior knowledge to observe the result, while the gene emergence peaks occurred during a cooling trend, the estimated species divergence and gene emergence occurred during LWP. As mentioned before, in this work, an LWP is a period with global surface temperature change greater than 0.5°C and lasting for 0.25 MY (i.e. 250 thousand years).

One existing explanation for species divergence when temperatures are dropping is that primate populations were forced to leave their original habitats and separated to different destinations (Morley *et al.* 2000; Begun *et al.* 2012). Zhou *et al.* (2014) studied the population size change of Himalayan species, although not specifically primates, during a long period of temperature change. The study observed speciation and population size reduction during a cooling phase, namely the penultimate glaciation, followed by population size recovery during a relatively warmer period. For primates, it is also possible that after the temperature began to locally rebound, the then primate-friendly temperature condition allowed the isolated primate populations to reproduce in larger numbers.

The recovery of population size and reproduction may be correlated to the discovery of emerged de novo genes. On seasonal primates living in environments with temperature change, previous research has revealed a positive correlation between warmer temperatures and their reproduction rates (Ziegler *et al.* 2000). One possible explanation behind this correlation is the abundance of food sources and fat deposition during warm periods allow greater energy investment towards reproduction (Randrianambinina *et al.* 2003). Another study showed that the spider monkeys live in warm forest environment suffered from reduced reproductive success after forest fragmentation, subsequently the loss of their warm and humid habitats, then the recovery of the forest helps recovery of their reproductive success (Galán-Acedo *et al.* 2019). Therefore, the emergence of new genes showed in the result of this study may be a result of a high rated reproduction period.

One general observation on de novo genes, not limit to primates, is de novo genes tend to have low expression level (Chen *et al.* 2015; Schlötterer 2015; Li *et al.* 2016; Schmitz *et al.* 2020) and are highly tissue specific (Schlötterer 2015; Schmitz *et al.* 2020), sometimes specifically related to brain function (Florio *et al.* 2017; Kalebic *et al.* 2018; Suzuki *et al.* 2018; Sun *et al.* 2020; An *et al.* 2023) and reproduction (Zhao *et al.* 2014; Lu *et al.* 2018). However, few papers have investigated the association between these characteristics and environmental change, especially in primates. A notable exception is the study by Papakostas *et al.* (2014), which reported an association between temperature conditions and gene expression level, as well as species evolution. This study showed that different environmental temperatures can affect gene expression in European grayling (*Thymallus thymallus*), resulting in variations in gene expression levels. Furthermore, this study suggests a link between gene expression level and gene adaptation, as well as the initial phases of evolution. Based upon the results presented in this research, it would be interesting to further investigate the relationship between de novo gene, gene expression, gene adaption in primate species, and environmental changes.

#### Primates prefer warm living environment

As a discussion on investigating de novo gene emergence in primates specifically, it may be worthwhile to consider certain characteristics of primate species themselves, such as their living environment. Hofreiter *et al.* (2015) showed that ancient nonhuman primates tended to live in conditions of high temperature and humidity. Another previous work showed that today, when the temperature is much cooler than 13 MYA, there still exists a positive correlation between primate species richness and mean annual temperature (Veeramah 2018). The tendency of primates to live in habitats closer to their original environmental conditions after climate change has also previously been studied with respect to humidity. Previous research has shown that after 8.1 MYA, when the climate continued to became drier and the aridity evidently intensified several times, many primate species tended to live in habitats with the highest humidity (Eronen and Rook 2004). One example is the distribution of genus *Macaca* approximately 6.8–7.2 MYA and 2.0–2.5 MYA was widespreading relatively humid areas in an overall arid area.

The distribution areas of the 31 nonhuman primates in this study are summarized and displayed in Table 1. This table shows that the 31 nonhuman primates with whole-genome sequences available are mostly found in Africa, South America, and Southeast Asia, which are comparatively warm areas on Earth. The distribution of nonhuman primates today shows a positive correlation with temperature (Veeramah 2018), which is also evident from the geographical location of the continents alone. It is possible that the results in this article have been biased by nonhuman primates that prefer warmer habitats. However, it should also be taken into account that both ancient and present nonhuman primates have been reported to prefer warmer environments, which may be considered as a characteristic of primate species themselves. When investigating the gene emergence in primates, this characteristic may need to be considered.

This is consistent with previous work (Elton and O’Regan 2014; Sayers *et al.* 2021; Cunningham *et al.* 2022). In Eocene and Miocene, primate species were more widely distributed. But at present, most of the nonhuman primates are indeed distributed in Africa, South America, and Southeast Asia. In North America, there are no known extant and native nonhuman primates. In Holocene, only one nonhuman primate species was found specifically in Europe, and it is now extinct (Elton and O’Regan 2014).

Note that for all 20 estimated species divergence times in the tree built from 32 primate species, not all of them are during LWPs. Also, this discovery depends on the accuracy of the divergence time and ranges. There are also genes estimated to have emerged at local cool periods within recent 10 MY. However, their total number is less than genes that emerged at LWPs. And these cooling divergence periods are inferred from the children clades on the timetree, with a 95% confidence interval.

### Other environmental conditions at primate gene emergence

As a discussion, geological events that affected and were affected by temperature can also be an interesting factor. In the recent 10 MY, one notable climate change is the enhancement of seasonal and dry conditions starting from approximately 9.2 MYA, possibly due to changes in precipitation (Barry *et al.* 2002). As the seasonality and drier climate become prevalent, types of plants, including forest and grass, have shifted starting from 8.1 MYA and formed open woodlands approximately 7.4 MYA (Barry *et al.* 2002). The intensification of the high-latitude glacial cycle occurred near 2.8 MYA has been suggested to be coincided with enhanced climatic diversity and drying conditions in Africa. The latter allowed for more diverse and open habitats near 2.9–2.4 MYA (Peter 2004). It is noteworthy that changes in temperature and climatic conditions affect not only primates themselves but also the plants that can form the environment of their habitats. The timing of these seasonal and aridity enhancements may coincide with the gene emergence can be an interesting subject for further exploration.

Also noteworthy are the plate movements and continental fragmentation, such as the fragmentation and counter-clock rotation of the Indian plate, which occurred approximately 10 MYA (Iaffaldano *et al.* 2011). Around this time point, few genes are estimated to emerge compared to adjacent time points. However, there is no sufficient data in this study to investigate the interesting question that whether the continental fragmentation approximately 10 MYA contributed to gene emergence. In the collection of 32 primate species, the only two pairs that have close divergence times are (1) species *Piliocolobus tephrosceles* and *Colobus angolensis*, diverged approximately 10.21 MYA and (2) two clades of 3 and 4 species diverged approximately 10.45 MYA. The first two species are found only in the central African continent. Out of the latter seven species, 4 are found only in the upper African continent and 3 are found only in Southeast Asia. Present study of continental plate movements considers the separation between the two plates occurred long before 10 MYA (Bosworth *et al.* 2005; Sands *et al.* 2017). Therefore these two divergences are unlikely to be associated with known continental fragmentation. Such a situation is the same for another interesting geological event, namely, the Great American Biotic Interchange approximately 4 MYA (Obando *et al.* 1996), when the formation of the Isthmus of Panama has increased the diversity of terrestrial mammals, but reduced the diversity of coastal and marine biota. In the data within this study, there are no primate species that are estimated to diverge around that time and lived in that region, therefore it is difficult to investigate the association between this event and gene emergence.

### Limitations and future work

This study is limited to the available sequence data of primates, characteristics of the 32 chosen primate species, and their adequacy for resolving specific problems in this study. The absence of unsequenced or extinct species in the timetree can influence the results, and such influence can be mitigated as more primate species are sequenced in the future. Estimation on ancient temperature is an ongoing subject of research and can be improved by data with higher accuracy and finer magnitudes to come. Some studies provided discussions on the methodology to estimate gene emergence time using species divergence time (Moyers and Zhang 2015), that this kind of method may lead to younger estimated gene emergence time. There are also other works suggesting that such concerns may be unnecessary (Domazet-Lošo *et al.* 2017). In this study, such potential bias was attempted to be mitigated by always accepting the earliest divergence time detected rather than the later ones, which resulted in a tendency for the genes to be estimated older than younger. The confidence of the results also depends on the 95% confidence interval of TimeTree database (Kumar *et al.* 2022) used in this study.

## Data Availability

The data sources of primate CDS, human protein sequences, timetree, and temperature curve are indicated in “Materials and methods.” The accession numbers of the 32 primate genomes, tables of human de novo genes, cancer-associated genes and random genes investigated in this study, as well as the scripts used to process the data and generate result figures, can be found at https://github.com/xlxlxlx/paleoclimate˙primate.
